# A crosstalk between autophagy and apoptosis in intracerebral hemorrhage

**DOI:** 10.3389/fncel.2024.1445919

**Published:** 2024-11-21

**Authors:** Moyan Wang, Xin Chen, Shuangyang Li, Lingxue Wang, Hongmei Tang, Yuting Pu, Dechou Zhang, Bangjiang Fang, Xue Bai

**Affiliations:** ^1^Department of Neurology, National Traditional Chinese Medicine Clinical Research Base, The Affiliated Traditional Chinese Medicine Hospital, Southwest Medical University, Luzhou, China; ^2^Institute of Integrated Chinese and Western Medicine, Southwest Medical University, Luzhou, China; ^3^Department of Emergency, Longhua Hospital Affiliated to Shanghai University of Traditional Chinese Medicine, Shanghai, China

**Keywords:** autophagy, apoptosis, cerebral hemorrhage, Bcl-2, Bax

## Abstract

Intracerebral hemorrhage (ICH) is a severe condition that devastatingly harms human health and poses a financial burden on families and society. Bcl-2 Associated X-protein (Bax) and B-cell lymphoma 2 (Bcl-2) are two classic apoptotic markers post-ICH. Beclin 1 offers a competitive architecture with that of Bax, both playing a vital role in autophagy. However, the interaction between Beclin 1 and Bcl-2/Bax has not been conjunctively analyzed. This review aims to examine the crosstalk between autophagy and apoptosis in ICH by focusing on the interaction and balance of Beclin 1, Bax, and Bcl-2. We also explored the therapeutic potential of Western conventional medicine and traditional Chinese medicine (TCM) in ICH via controlling the crosstalk between autophagy and apoptosis.

## 1 Introduction

Stroke, always a magnet for medical researchers, causes permanent brain damage, serious disability or even death (Diener and Hankey, 2020). Hemorrhagic stroke is more fatal than ischemic stroke, and the former is subdivided into intracerebral hemorrhage (ICH) and subarachnoid hemorrhage (SAH) (Feigin et al., 2009). The incidence and prognosis of ICH vary with races and socioeconomic statuses. Asians suffer from a higher susceptibility to ICH than other ethnic groups (Johnson et al., 2016). Within the past four decades, the incidence of ICH has sharply declined in high-income countries, but almost doubled in low-and middle-income countries (Feigin et al., 2009). These fluctuations become more evident within the past two decades (Flaherty et al., 2007; Khellaf et al., 2010; Islam et al., 2008; Huhtakangas et al., 2011). A decreasing trend may be attributed to the rapid boom of medical resources, while a rising trend to the wide use of anticoagulant medications like warfarin (Lovelock et al., 2007).

Generally, ICH results in primary and secondary injuries. The primary injury happens when cerebral blood vessels abruptly rupture, leading to hematoma that exerts mechanical damages on adjacent tissues and a swift increase in intracranial pressure (Zhao et al., 2018). Red blood cell fragments and blood flow back into the brain cause secondary excitotoxic and cytotoxic brain injuries, like neuronal apoptosis, inflammatory response, oxidative stress, mitochondrial dysfunction and blood–brain barrier (BBB) destruction (Zhou et al., 2014; Chen et al., 2018). Regional ICH in the thalamus, cortex, brainstem, and areas beneath the cerebellar tentorium brings with severe motor, sense and language dysfunctions. Moreover, ICH patient also demonstrate nonspecific symptoms, such as headache, nausea, and meningeal irritation, all detrimental to the quality of life (Qureshi et al., 2001).

**Figure 1:**
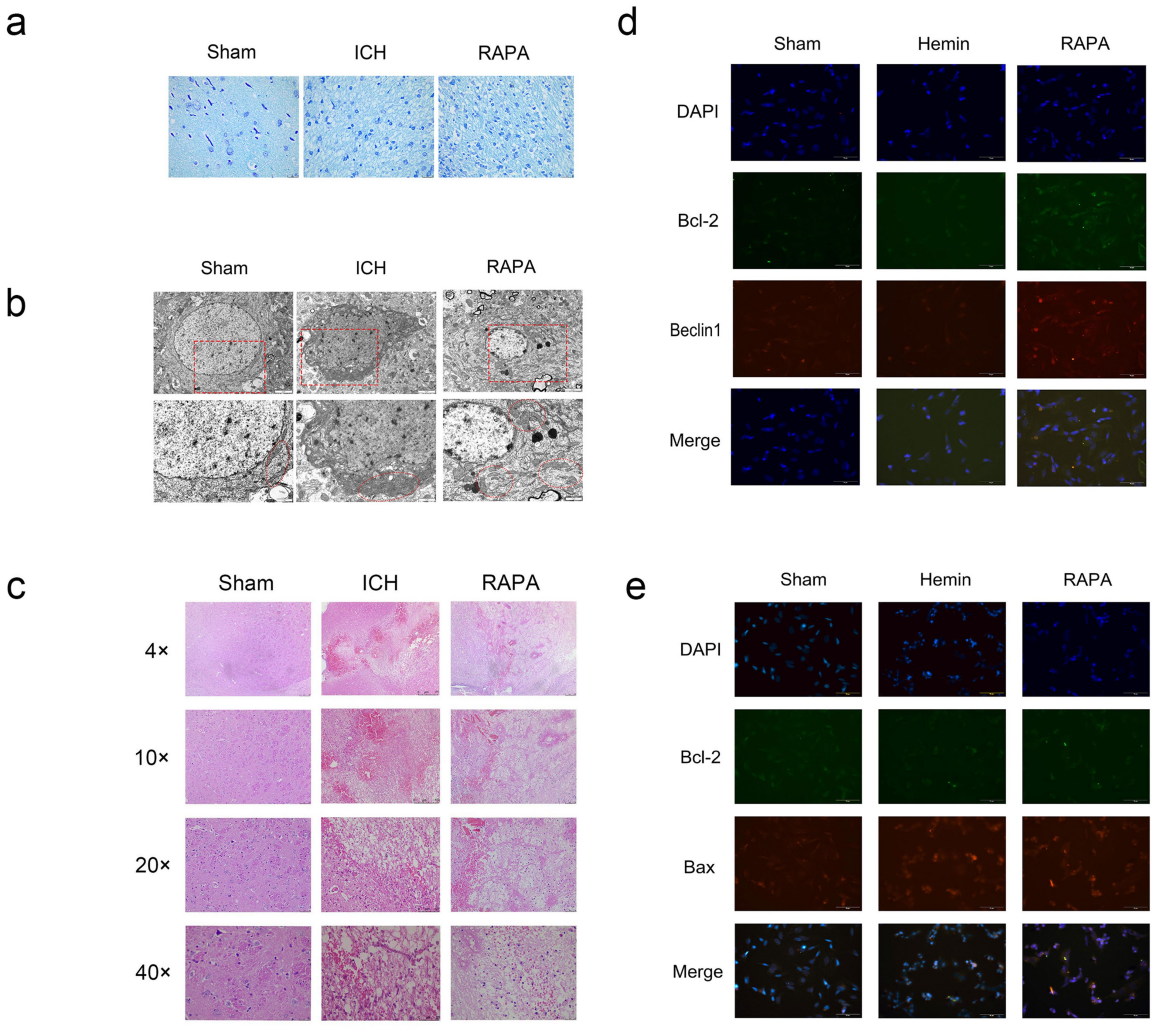
Rat intracerebral hemorrhage model induced by collagenase: the sham group was untreated, the Ich group was a simulated intracerebral hemorrhage group, and the Rapa group was treated with rapamycin. Sh-Sy5Y human neuroblastoma cells induced by hemin iron. The sham group was untreated, the hemin group was a simulated intracerebral hemorrhage group, and the Rapa group was treated with rapamycin. (a) Nissl staining (400×). (b) Electron microscopy (8,000×, 15,000×) (c) Hematoxylin–eosin staining (40×, 100×, 200×, 400×). (d) *In vitro* immunofluorescence Beclin1, Bcl-2 double staining results (400×). (e) *In vitro* immunofluorescence Beclin1, Bax double staining results (400×).

Massive apoptotic cells in the brain at post-ICH have been observed in *in vivo* models (Duan et al., 2020; Qureshi et al., 2001; Figure 1). Consistently, a great number of apoptotic cells are found around the site of hematoma in patients with spontaneous ICH (Qureshi et al., 2003). Ubiquitinated proteins and p62 accumulate in neurons from nearly all regions of the autophagy-defective brain, generating inclusion bodies age-dependently growing in size and quantity (Lee et al., 2010). Neurodegenerative diseases progress with an accumulation of autophagic vacuoles (Fleming et al., 2011). Cathepsin D is a lysosomal enzyme for regulating the accumulation of autophagic vacuoles (Araki et al., 2006). Produced in neurons and astrocytes rapidly following the brain hemorrhage, Cathepsin D shows the maximal activity at 1 week, and keeps continuous hyperactivity for 4 weeks or longer (He et al., 2008). Upregulation of autophagy-related proteins Beclin 1 and LC3 and downregulation of anti-apoptotic protein Bcl-2 at post-ICH prove that autophagy is activated by ICH (Shen et al., 2016). Some studies indicate that autophagy promotes cell survival (Carloni et al., 2008), whereas other research suggests that inhibiting autophagy may enhance survival probability (Puyal et al., 2009; Wang and Zhang, 2017)^.^ A long-term inhibition on autophagy is harmful to cell viability, while a short-term suppression may be acceptable (Smith et al., 2011). Mice lacking Atg7 in the central nervous system (CNS) present behavioral problems and die 28 weeks after birth, suggesting the health-threatening effects of autophagy deprivation (Komatsu et al., 2006). On the contrary, toxic proteins can be degraded by autophagy to favor cell survival and maintenance. Clinical studies have yet to explore the treatment of cerebral hemorrhage through the modulation of autophagy; however, acupuncture has been shown to concurrently reduce both autophagy and apoptosis (Yao and Zou, 2022). Consequently, autophagy represents a promising avenue for future research aimed at mitigating damage following cerebral hemorrhage (Chen et al., 2024).

## 2 Critical roles of autophagy and apoptosis in neurological outcomes of ICH

**Figure 2:**
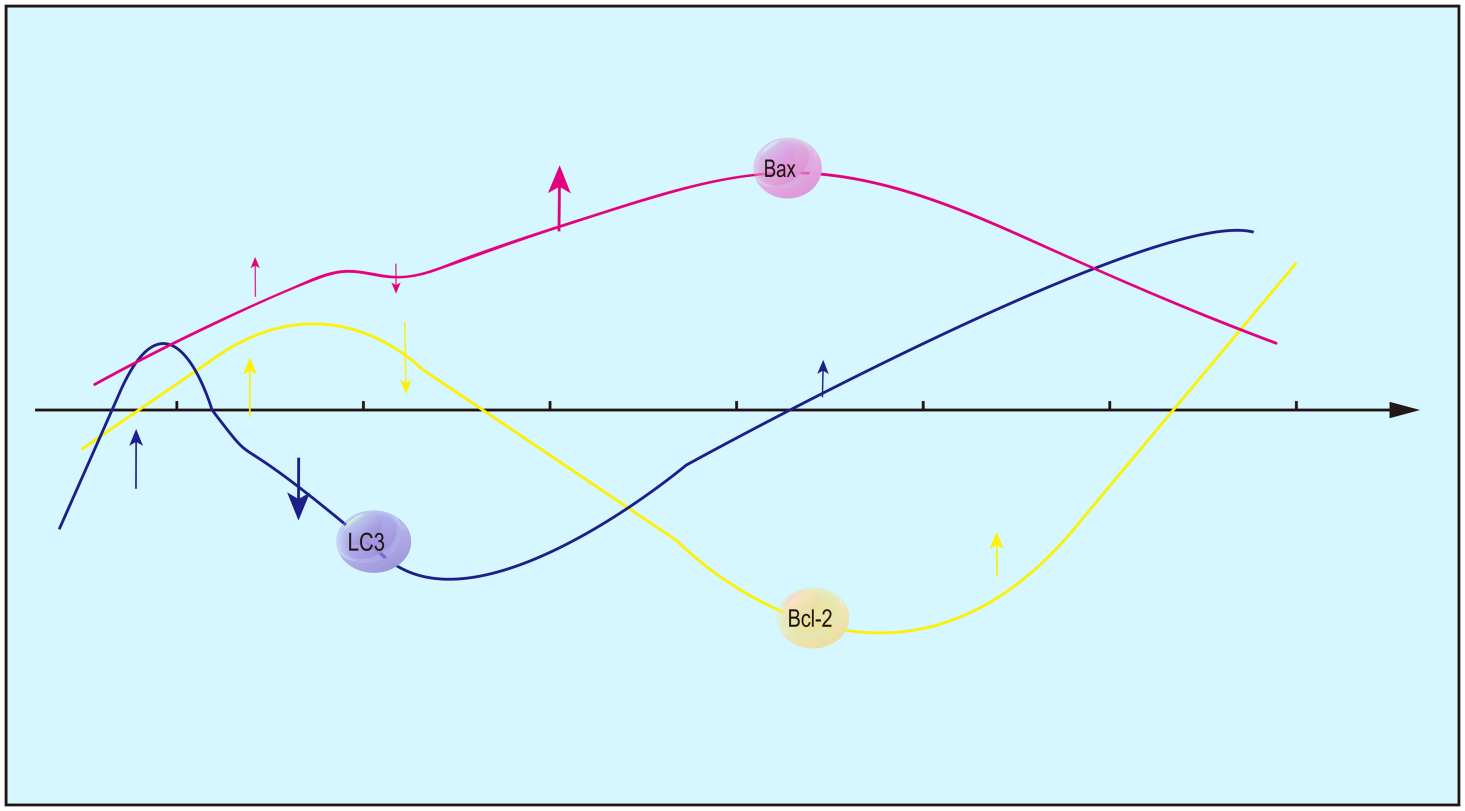
Changing trends in the expression of Bcl-2, Bax and LC3-II/LC3-I after ICH.

Expression levels of Bcl-2 and Bax peak at 12 h and 1 day following ICH (Figure 2), respectively (Xuefei et al., 2011). At 6 h of ICH, blood osmosis and mechanical injury are examined in the brain sections, while nerve cell edema is absent and TUNEL-positive cells remain in a low proportion. Moreover, expression level of Bcl-2 decreases sharply in the brain sections after ICH, but increases to the baseline at 12 h and peaks at 3 days (Xiu'e and Liangqun, 2010). Besides apoptosis, autophagy involving Bcl-2 and Bax is speculated to participate in the acute phase of ICH, and the Bcl-2/Bax ratio can be used to infer changes in autophagy. Consistently, fluctuations in LC-3II/LC-3I surrounding hematomas and an increase within 6 h of ICH are indicative of autophagy in rats (Huaxian et al., 2003).

Investigator (Lee et al., 2015) utilized Western Blot analysis to determine that Bcl-2 expression peaked at 12 h following cerebral hemorrhage, while Bax expression peaked at 1 day. Pathological findings (Yanling et al., 2004) indicated that 6 h post-intracerebral hemorrhage (ICH), there was evidence of blood infiltration and mechanical damage, but no neuronal edema was observed. The number of TUNEL-positive cells was one-eighth of the peak value. However, in comparison to the sham group, Bcl-2 expression significantly decreased, only to rise above the average level of the saline group after 12 h, reaching its maximum at 3 days. This suggests that, in the acute phase, there are cellular responses beyond apoptosis, and it is plausible that Bcl-2 plays a role in the regulation of autophagy in conjunction with Bax. The Bcl-2/Bax ratio not only serves as an indicator of apoptosis but may also influence autophagy through alterations in this ratio, as evidenced by findings from a separate study (Duan et al., 2017). Furthermore, the autophagy marker LC-3/LC-3I was observed in rats with intracerebral hemorrhage (ICH) at 6 h post-injury, showing a significant recovery between 72 h and 7 days. Additionally, the study indicated that the observed autophagy may be indicative of excessive autophagic activity. By comparing autophagy agonists and autophagy inhibitors, it was demonstrated that excessive autophagy within 6 h led to an increase in the cleavage of Caspase-12, while inhibition of autophagy led to a reduction in damage.

Autophagy and apoptosis are normal physiological activities both involved in self-repair. To curb a pathological state, efforts can be made for increasing the possibility of autophagy without stimulating apoptosis. Not only rapid increases in expression levels of Bax and Bcl-2 following ICH respond to self-repair or self-clearance mechanism. Autophagy-induced apoptosis provides various effects. Inhibiting autophagy lowers apoptosis in rats with transient ischemic attack (Huang et al., 2020). On the contrary, an excessive autophagy during cardiac arrest and resuscitation causes neuronal apoptosis and worsens brain damage (Youle and Narendra, 2011). These diametrically opposed results may be attributed to the dual functions of autophagy that can actively promote apoptosis by removing damaged cells, maintaining tissue homeostasis, and eradicating harmful cells. Removal of damaged mitochondria during autophagic processes also prevents cell apoptosis caused by mitochondrial dysfunction (Green and Llambi, 2015; Ebrahimi-Fakhari et al., 2012). Autophagy and apoptosis have a blurred boundary, and it is challenging to quantify what a level of autophage can contribute to apoptosis.

## 3 Regulators of autophagy and apoptosis

**Figure 3:**
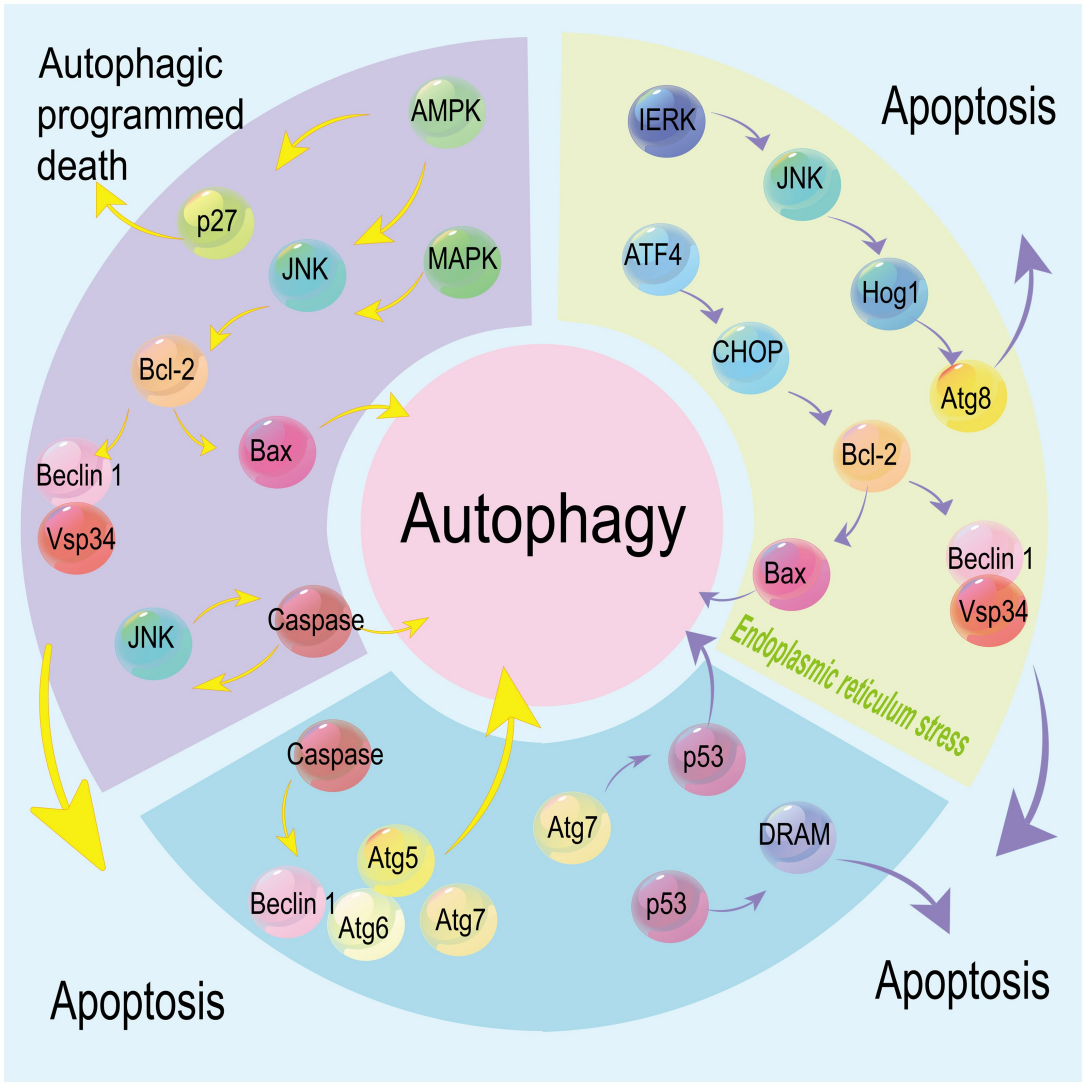
Multiple factors and signaling pathways mediate both autophagy and apoptosis. Under endoplasmic reticulum (ER) stress, autophagy regulated by the IERK/JNK/Hog1 axis via stabilizing Atg8 co-exists with apoptosis regulated by the ATF4/CHOP axis. p53 is involved in the regulation of autophagy and apoptosis mediated by DRAM and Atg7, respectively. Caspases mediate the cleavage of Beclin 1, Atg6, Atg5 and Atg7, linking apoptosis with autophagy signaling pathways. JNK is activated by MAPK and AMPK to trigger caspase-induced cell death, while phosphorylated AMPK also induces autophagy by stabilizing p27. Downstream Bcl-2 is involved in apoptosis and autophagy via mediating Bax and Beclin 1.

Multiple factors and signaling pathways regulate both autophagy and apoptosis, and their upstream factors may be involved in the crosstalk between autophagy and apoptosis (Figure 3). For instance, downregulation of autophagy factor Atg5 inhibits both autophagy and apoptosis (Dong et al., 2019). Atg6 serves as a switch that connects apoptosis and autophagy, and its downregulation increases the susceptibility to TRAIL-induced cell death, suppresses apoptosis, and promotes autophagy (Cho et al., 2009). P53 is either activated or inhibited in autophagy, and also an initiator of apoptosis. DRAM links autophagy to p53, and loss of Atg7 exacerbates p53-dependent apoptosis (Crighton et al., 2006; He et al., 2013). Besides, p53 alters the Bcl-2/Bax ratio and the state of Bax overweighing Bcl-2, thereafter inducing apoptosis (Yan et al., 2021). The binding ability of p53 to Bax and Bad is enhanced by phosphorylation of Bcl-2 at Ser70, a process necessary for its anti-apoptotic function (Maiuri et al., 2007). Also, phosphorylation of Bcl-2 blocks the interaction between Bcl-2 and pro-apoptotic protein BH3, resulting in the apoptotic cell death when the autophagy activated by phosphorylated Bcl-2 no longer supports cell survival (Wei et al., 2008). AMPK breaks the balance between autophagy and apoptosis, and tilts it to the former. Additionally, phosphorylation of AMPK activates autophagic cell death (Liang et al., 2007; Kang et al., 2011). Caspase-dependent cell death is triggered by the cleavage of Beclin 1 and Atg7. Caspase-8 inhibits cell death and thus alleviates the severity of illness (Shimizu et al., 2004; Yu et al., 2004; Mizushima and Komatsu, 2011).

The MAPK8/JNK1/ERK signaling pathway or phosphorylation of DAPK stimulates autophagy by breaking the balance between Bcl-2 and Beclin 1 (Zalckvar et al., 2009; Fang et al., 2019). Besides autophagy, ER stress is another event triggering apoptosis. Stimulated by the unfolded protein response, ER membrane-resident stress sensor IRE1 interacts with TRAF2 and then activates JNK to initiate cell apoptosis. ER stress-induced autophagy can be activated by the IRE1/JNK/Hog1 and ATF4-associated signaling pathway through enhancing the ATG8/LC3 stability (White, 2016). Regulated by the ATF4/CHOP axis, an extreme ER stress stimulates cell apoptosis through downregulating Bcl-2 and upregulating Bax (Maiuri et al., 2007). The dynamic interaction of LC3B with Cav-1 and Fas controls cigarette smoking-induced apoptosis (Chen et al., 2010).

## 4 A subtle balance between apoptosis and autophagy

### 4.1 Bcl-2/Bax/Beclin 1

Members of the Bcl-2 family regulate cell death, and are involved in both apoptosis and autophagy (Gross et al., 1999). Functionally, a part of Bcl-2 family members is anti-apoptotic, and the remaining are pro-apoptotic. The antagonism between two camps determines the threshold of cell fate, serving as a common phenomenon in human beings. Members of the Bcl-2 family initiate or prevent the release of apoptotic factors via regulating the mitochondrial integrity by forming homodimers and heterodimers (Uren et al., 2017; Westphal et al., 2014). They also participate in the non-apoptotic cell death dependent on autophagy genes after knocking out the apoptotic genes BAX/BAK (Shimizu et al., 2004).

**Figure 4:**
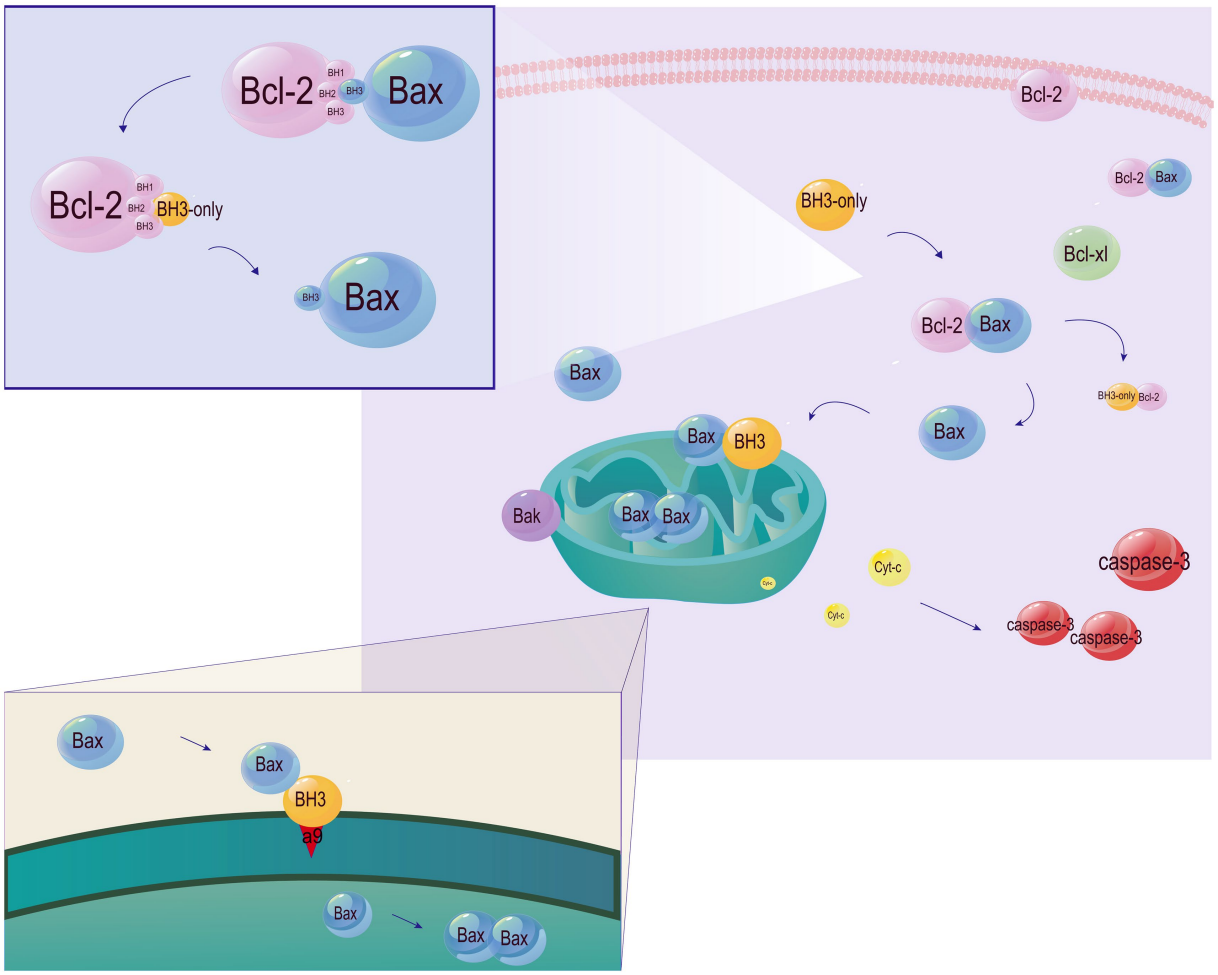
Involvement of the Bcl-2 family members in apoptosis. Under physiological conditions, Bak and Bax are localized in the outer mitochondrial membrane and mitochondria, respectively. Translocation of Bax to the cytoplasm increases the sensitivity to apoptosis. Bcl-2 and Bcl-xL are localized in the membrane and cytosol, respectively. Both of them control the translocation of Bax/Bak. BH3-only proteins are responsible for transmitting cell death signals to other members of the Bcl-2 family. Relying on the BH3 domain, BH3-only proteins bind to anti-apoptotic members of the Bcl-2 family and inactive them, thus indirectly activating Bax/Bak. Moreover, α9 released by BH3-only proteins from a hydrophobic groove directly activates Bax by inserting into the MOM. Bax monomers then form dimer units, self-assemble into oligomers and generate pores in the MOM, leading to the caspase cascade by the release of cytochrome C.

Through aggregation or segregation, the Bcl-2 family regulates cell apoptosis (Figure 4). The four conserved BH domains determine biological functions of the three subfamilies of Bcl-2. Generally, there is a homologous sequence shared by all the four BH domains of most anti-apoptotic members in the Bcl-2 family, while homologous sequences are only observed in the BH3 domain of pro-apoptotic members (Cohen et al., 2020). BH3-only proteins interact with the BAX N-terminal trigger site, and stimulate the translocation of cytoplasmic Bax to the mitochondrial outer membrane (MOM), leading to the altered mitochondrial outer membrane permeabilization (MOMP) and mitochondrial dysfunction. The formation of heterodimers by anti-apoptotic and pro-apoptotic Bcl-2 family members prevents Bax-induced MOMP alteration and mitochondrial dysfunction through trapping the exposed BH3-domains of pro-apoptotic members in the surface groove. The inhibited release of soluble pro-apoptotic factors (e.g., cytochrome c) from the intermembrane space ultimately prevents caspase-mediated apoptosis (Garner et al., 2019; Subburaj et al., 2015). In addition, BH3-only proteins directly or indirectly activate Bax/Bak by binding to Bcl-2 and inactivate it (Westphal et al., 2014; O'Neill et al., 2016). Bax and Bak cause apoptosis by generating pores or binding to BH3-only proteins (Uren et al., 2017; Chen et al., 2005). Cleavage products of the anti-apoptotic factors Bcl-2, Bcl-xl, and Mcl-1 produced by Caspase-9 effectors also cause apoptosis (Mizushima and Komatsu, 2011).

Beclin 1, a mammalian positive regulator of autophagy in the Bcl-2 family, contains a BH3 domain, a central coiled-coil domain and an evolutionarily conserved domain. Serving as an orthologue of yeast Atg6, Beclin 1 is considered to mediate autophagy and restore the autophagy activity having been destroyed by Atg6 (Kihara et al., 2001). During the development of *Caenorhabditis elegans*, loss of Beclin-1 increases caspase-dependent apoptosis and the number of apoptotic bodies (Takacs-Vellai et al., 2005). In as early as 2001, the Beclin 1 homolog of yeast Vps30 was found to assemble two complexes of Vps15 and Vps34 (Salminen et al., 2013). Beclin 1 interacts with Vps34 through the central coiled-coil domain and evolutionarily conserved domains, and the latter forms phosphatidylinositol-3-phosphate (PI3P) by phosphorylating phosphatidylinositol (Feng et al., 2007; Konishi et al., 2012). In early-stage phagosomes, Beclin 1, Rac1 and PI3P binding protein containing the FYVE domain assemble into the class III PI3K complex. Induction of the PI3K inhibitor 3-Methyladenine (3-MA) or knockout of Beclin 1 identically slows down the process of phagocytosis, indicating that phagocytosis is dependent on Beclin 1 and the activity of PI3K (Funderburk et al., 2010). The Beclin 1-Vps34 complex can also invite other partners to form new complexes. Specifically, interacting partners of the Beclin 1-Vps34 complex include Atg14L, UVRAG and Rubicon; while unstable or transient binding partners compose of Bif-1, Ambra 1, VMP1, nPIST, Rab5, ICP 34.5, and inositol 1,4,5-triphosphate receptor (IP3R). These important complexes are involved in all stages of autophagy to mediate the formation or maturation of autophagosomes, endocytic transport and autophagy signaling pathway. Endogenous Bcl-2 is able to interfere with the binding of Beclin 1 to Atg14L, UVRAG or Rubicon (Feng et al., 2007).

The BH3 domain of Beclin 1 binds to members of the Bcl-2 family. As a result, the apoptosis regulator Bcl-2 also mediates autophagy by interacting with Beclin 1 (Figure 4). Anti-apoptotic proteins encoded by cells and viruses effectively bind to the BH3 domain (He and Levine, 2010). The binding of Bcl-2 to Beclin 1 is enhanced in nutrient-rich conditions where autophagy is inhibited, and decreases in nutrient-deficient conditions where autophagy is stimulated. In addition, Bcl-2 impairs the interaction between Beclin 1 and Vps34 and reduces Beclin 1-associated Vps34 kinase activity, thereby potentially sequestering Beclin 1 away from the autophagy-inducing class III PI3K “core complex” (Pattingre et al., 2005). Loss of Beclin 1 suppresses autophagy, leading to cell death under nutrient deprivation and other stress conditions. The Bcl-2-Beclin 1 complex is critical for stimulus-induced autophagy in mammalian cells. Phosphorylated Bcl-2 inhibits autophagy by directly interacting with the BH3 domain of the ER-targeted Beclin 1. Approaches through phosphorylating Bcl-2 or binding to it interrupt its interaction with Beclin 1, thereby promoting autophagy (He et al., 2012; Danial and Korsmeyer, 2004). Members of the Bcl-2 family also influence other cellular processes like glucose homeostasis, cell cycle progression, calcineurin signaling, and transcriptional inhibition of p53 (Shimizu et al., 2004). Stimulated by starvation, phosphorylation of Bcl-2 at Thr69, Ser70, and Ser87 dislocates it from Beclin 1 and then induces autophagy (Wei et al., 2008). Replacement of these sites with alanine on Bcl-2 is reported to reduce the autophagy and glucose homeostasis in mouse skeletal muscles after exercise (Pattingre et al., 2009).

**Figure 5:**
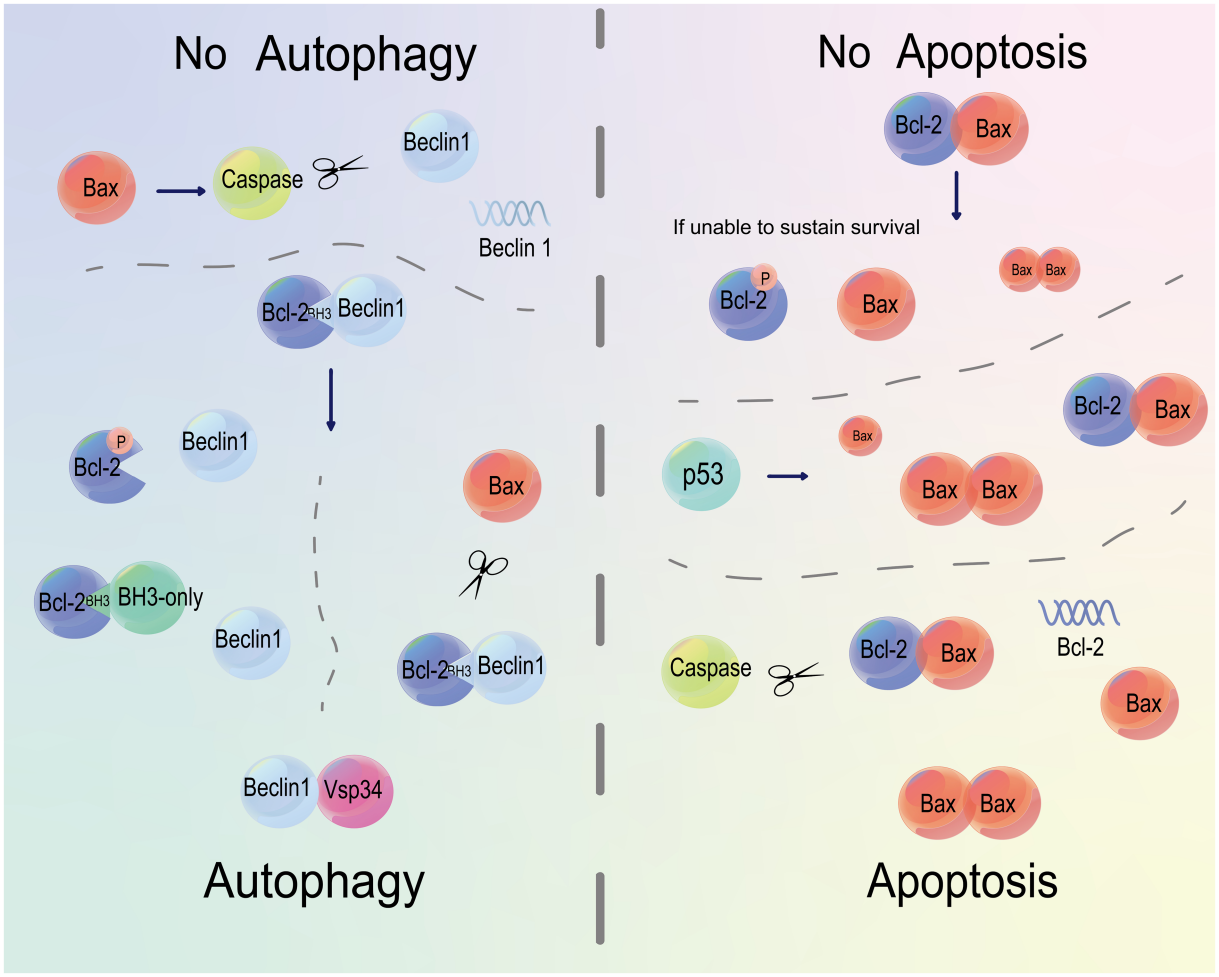
Bcl-2 and Bax regulate both apoptosis and autophagy. The binding of Bcl-2 to Beclin 1 or caspase cleavage of Beclin 1 inhibits autophagy. Phosphorylation of Bcl-2 or occupation on its BH3 domain activates autophagy by triggering the binding of Beclin 1 to Vsp34. Moreover, Bax can cleave Bcl-2 and Beclin 1. The binding of Bax to Bcl-2 prevents apoptosis. A decline in phosphorylated Bcl-2 by Bax cleavage below a threshold to maintain survival eventually causes apoptosis. p53 disrupts the balance between Bcl-2 and Bax, and orients this balance to the latter, leading to apoptosis. Caspases serve as regulators of apoptosis by cleaving Bcl-2.

Bax also serves as a regulator of Beclin 1 (Figure 5). Bax/caspase induction cleaves Beclin 1 and inhibits autophagy. Pan-caspase inhibition stabilizes Beclin 1 and rescues Bax-mediated autophagy inhibition in the presence of Bax, suggesting the role of Beclin 1 as a caspase substrate (Yu et al., 2004). Overexpression of Bax does not increase the anti-apoptotic ability of cells, but inhibits autophagy through downregulating Beclin 1 via caspase cleavage. On the contrary, in cells equipping a strong anti-apoptotic capacity, overexpression of Bcl-xL or induction of caspase inhibitor stimulates autophagy by breaking the Bcl-xL-Beclin 1 interaction via Bax or Bcl-2 (Yong et al., 2010). Collectively, an orientation towards Bcl-2 blocks the interaction between Bcl-2 and Beclin 1 and thereafter increases autophagy.

### 4.2 Bcl-2/Bax/Beclin 1 regulate apoptosis and autophagy

Bcl-2 deficiency is not the only determinator for apoptosis, which also requires for the participation of Bax (Wall et al., 1999). The Bcl-2/Bax ratio is a predictive marker for cell sensitivity to apoptosis (Weidong and Zhengtang, 2007). Mathematical modeling of apoptosis shows that the ratio of pro-apoptotic and anti-apoptotic members of the Bcl-2 family serves as a hub to control apoptosis by directly determining the opening degree of mitochondrial ion channels of the outer membrane. Bcl-2 induces apoptosis by Bax homodimerization, but counters apoptosis by Bax heterodimerization. It is reported that Bax homodimerization/heterodimerization predicts cell fate. Cells containing 50% of Bax in heterodimerization of Bcl-2 and Bcl-xL are resistant to apoptosis, while those containing 80% of Bax in homodimerization are sensitive (Selvakumaran et al., 1994). The anti-apoptotic capacity of the Bcl-2 family largely relies on the expression of Bcl-2 and the formation of homodimers/heterodimers with Bcl-2 (Figure 4). Homodimerization of Bax triggers cell death, while heterodimerization of Bcl-2 to Bax favors cell viability. Generally, the Bcl-2/Bax ratio is intrinsic under normal physiological conditions, but altered by external stimuli like the p53-dependent cell death signaling (Crawford et al., 1998; Oltvai and Korsmeyer, 1994).

Overexpression of Bcl-2/Bcl-xL leads to autophagy, while overexpression of BH3-only proteins fails to induce non-apoptotic cell death in cells with loss of Bax/Bak. Hence, the balance between Bcl-2/Bcl-xL and Bax/Bak determines the status of autophagy (Luo and Rubinsztein, 2010). Caspases not only act as final executioners of apoptosis, but also participate in maintaining the above balance. Bax-induced caspase activation results in Beclin 1 cleavage and then inactivates autophagy. Pan-caspase inhibition remarkably stabilizes Beclin 1 and rescues from Bax-induced autophagy inhibition, indicating that Beclin 1 is the substrate of caspase. Autophagosomes are reduced following Bax-dependent cleavage of Beclin 1 or binding of Bcl-xL to Beclin 1. It is found that co-transfection of Bcl-xL and Bax normalizes the number of autophagosomes. Overexpression of Bax causes different outcomes of autophagy based on the anti-apoptotic state. Bax either reduces autophagy flux in cells without an increased anti-apoptotic ability by downregulating Beclin 1 via caspase cleavage, or induces autophagy in cells presenting an increased anti-apoptotic ability by overexpressing Bcl-xL or treatment of caspase inhibitors (Yu et al., 2004).

### 4.3 Balance among Bcl-2, Bax, and Beclin 1

Phosphorylation of Bcl-2 dissociates the Beclin 1-Bcl-2 complex and thus triggers autophagy. Bcl-2 also binds to Beclin 1 and breaks the Beclin 1-Vps34 complex, although it is not usually required for Bcl-2/Bcl-xL-induced suppression of autophagy. Compared with the Beclin 1-Vps34 complex, a multiprotein complex involving the Bcl-2/Bcl-xL, Beclin 1, and Vps34 inhibits autophagy by reducing the Class III PI3K activity of Vps34 (Pattingre et al., 2005). Loss of Bcl-2 results in cell death by inducing an excessive activity of autophagy, indicating that Bcl-2 functions as an interceptor to prevent autophagy-induced processes necessary for apoptosis (Yee et al., 2009). In cell apoptosis, Bcl-2 functions as a suppressor by preventing Bax homodimerization. During autophagy, Bax functions conversely to Bcl-2. Bax inhibits autophagy by promoting caspase-3-induced dissociation of Beclin 1 to break the interaction between Vps34 and Beclin 1. Besides, Bax stimulates autophagic flux influenced by the mitochondrial perturbation, and inhibits Bax-induced autophagy to further suppresses apoptosis. It is indicated that mitochondrial autophagy decides the cell fate (Maiuri et al., 2007). Members in the BH3-only subgroup of the Bcl-2 family, such as Bad, induce autophagy by acting on the BH3 domain and BH3 domain-binding groove of Bcl-2/Bcl-xL to impair the interaction between Bcl-2/Bcl-xL and Beclin 1 (Maiuri et al., 2007). Directly or indirectly, BH3-only proteins induce apoptosis through controlling the mitochondrial permeability of pro-apoptotic Bcl-2 family members (Oltersdorf et al., 2005). After a deep mining of the mechanism of apoptosis, BH3 mimetics of ABT-737 and its oral bioavailable analog Navitoclax have been designed and developed to stimulate cell apoptosis by targeting anti-apoptotic factors like Bcl-2, Bcl-xL and Bcl-w (Lee et al., 2015). Considering their architectures, we believed that BH3 mimetics are also promising mediators of cell autophagy.

### 4.4 Interaction between apoptosis and autophagy in the neurological damage of ICH

Autophagy is an initiator and competitor of apoptosis, as well as a collaborator to push cells into the abyss of death. Only Beclin 1 in the Bcl-2 family regulates autophagy, and is regulated by Bcl-2 and Bax. Apoptosis and autophagy hold differing sequences in cellular component degradation. During the process of apoptosis, the cytoskeleton is degraded in the early stage, and the organelles still exist in the late stage. In contrast, degradation of organelles occurs in the early stage of autophagy and the cytoskeleton remains in the late stage (Levine and Yuan, 2005). After triggering the apoptotic pathway, cells that are no longer needed commit suicide by shrinking and condensing the cytoplasm, organelles and chromatin. Eventually, cytoskeleton and nuclear proteins simultaneously undergo apoptosis (Slee et al., 2001). Macroautophagy is the major type of autophagy, in which portions of the cytoplasm are sequestered within autophagosomes and delivered to lysosomes for bulk degradation (Klionsky and Emr, 2000; Giricz et al., 2012). Lysosomes formed by autophagic vacuoles or autophagosomes during autophagy are responsible for engulfing the wrapped cytoplasm, organelles or proteins to synthesize essential molecules required for energy metabolism (Chen et al., 2010).

Autophagy, responded by a short-term stimulation, initiates to remove garbage organelles and particularly apoptosis-sensitive mitochondria. Through preventing the aggregation of lethal proteins and selectively removing pro-apoptotic proteins in the cytoplasm (e.g., caspase activators), autophagy shoulders a mission to prevent cell death. Therefore, autophagy, as a protective measure of self-rescue, reduces the sensitivity to apoptosis via eliminating harmful cellular components (Mariño et al., 2014; Bursch, 2001). However, a long-term stimulation uncovers the preface of programmed cell death (Gutierrez et al., 2014). Autophagy-dependent cell death, caused by excessive autophagy, is strictly defined as cell death caused by an autophagic flux. Presence of autophagosomes in dying cells, which may be probably caused by the blockage of autophagic flux (e.g., inhibition of caspase-10), is inconclusive of autophagic cell death (Mariño et al., 2014). Occasionally, blockage of the autophagosome-lysosome fusion increases autophagic flux, but inhibits autophagy. Current findings suggested that the positive expression of the autophagy substrate p62 is a reliable marker for autophagy-dependent cell death (Fricker et al., 2018). Instead of mutually exclusive, apoptosis and autophagy can both occur in the same type of cells. Initially stimulated by deprivation of growth factors, toxic drugs or hypoxia, autophagy usually mounts its stage prior to apoptosis, as an adaptive strategy. Once autophagy is powerless to counteract the long-term intolerable stress, apoptosis immediately joins to fight against unwanted cells (Yousefi et al., 2006; Kroemer et al., 2010). During their collaboration, autophagy assists to remove damaged components that are resistant to apoptosis (Lamparska-Przybysz et al., 2005).

Recovery of neurological function is closely linked with autophagy and apoptosis. Neuronal apoptosis in isolated brain regions results in the dysfunction of neural circuits, eventually leading to a selective loss of vulnerable neurons (Brown, 2021; Baron et al., 2014). Notably, neuronal apoptosis in the hematoma at post-ICH induces peripheral neuron damage, further aggravating neurological deficits. Through removing neuronal debris and reducing protein aggregation, autophagy exerts a protective role to promote neuron regeneration in patients with neurodegenerative diseases (Li et al., 2020; Martini-Stoica et al., 2016). Controversially, ICH rats overexpressing miR-146a present recovery of neurological function but a low profile of autophagy (Huan et al., 2020). The role of autophagy in ICH remains to be further analyzed.

## 5 ICH-associated autophagy and apoptosis

Autophagy and apoptosis of multiple types of brain cells, such as neurons, endothelial cells and microglia following ICH, pose vital influences on the prognosis. The formation of hematoma following ICH causes primary mechanical damages to surrounding tissues, and secondary hypoxic–ischemic toxicity further aggravates the mechanical damage.

Owing to reduced blood perfusion and hypoxia post-ICH, an ischemic penumbra is visualized around the ischemic core, with neurons are functionally impaired with lower activities, but not yet infarcted. Preserving tissue in the ischemic penumbra has therefore become the top priority (Back, 1998). Caspase-3 activation is initially observed in the ischemic penumbra after 3 h of ischemia–reperfusion injury (IRI), followed by the detection of its positive expression in the ischemic core at 24 h. On the contrary, the activity of caspase-3 remains low after the occlusion in the whole brain (Manabat et al., 2003). Suppression on apoptosis using caspase inhibitors offers a narrow treatment window, presenting efficacy on transient cerebral ischemia rather than the global cerebral ischemia (Li et al., 2000). Apoptosis is therefore believed as a rapid response in the local ischemic areas experiencing re-perfusion. The proportion of apoptotic cells is theoretically higher in the hypoperfused penumbra than in the ischemic core. Rapamycin is an autophagy activator that rescues hippocampal neuron death caused by global cerebral ischemia, suggesting that autophagy can prevent non-apoptotic cell death under certain conditions (Hwang et al., 2017). Autophagy abolition alone is sufficient to drive protein aggregation, leading to neuronal death and neurodegenerative diseases.

GFP-LC3 is an imaging marker on the autophagosomal membrane. Under physiological conditions, autophagy is barely detected in the brain (Mizushima et al., 2004). It is only detectable in the cortex and hippocampus following brain injuries (Weis et al., 2014). Following the stimulation from ischemia, autophagy increases in the early stage, particularly in cortical neurons, and gradually decreases alongside the weakening of lysosomes and aggravation of ischemic injuries (Liu et al., 2019). Just the increase in autophagosomes may result in an ineffective accumulation or even neurotoxicity. Ischemic injuries may be treated through interventions to keep a dynamic balance between autophagosomes and lysosomes (Menzies et al., 2017). In the brain, neurons are responsible for autophagosome biogenesis and trafficking. Due to their extreme sensitivity to autophagy, neurons also die with excessive autophagy (Benito-Cuesta et al., 2017).

Initially, apoptosis mainly occurs in neuronal cells following stroke (Manabat et al., 2003). After induction of excitotoxicity, p53, as a regulator of programmed cell death, triggers neuronal damages in sympathetic neurons after mitosis (Sakhi et al., 1994). Neurons connect and communicate with each other through synapses. When a neuron fails in response to a synaptic input or output, the connected neuron dies. In cases of ICH, neuronal death in the ischemic area results in the demise of neurons in the surrounding (known as the selective neuron loss) and remote regions (secondary neurodegeneration) (Brown, 2021; Baron et al., 2014). Neuronal apoptosis in the ischemic cerebral area may involve surrounding neurons, which is usually seen in ischemic stroke and Alzheimer’s disease.

Microglia are a type of immune cells that polarize in response to various brain injuries, including non-regional damages to neurons. Stimulated by excitotoxicity, sustained ischemia or inflammation, neurons induce the phagocytosis of microglia through releasing “find-me” and “eat-me” signals or binding to opsonins, like complement components C1q and C3b (Brown, 2021). Erythrolysis following ruptures of cerebral blood vessels triggers autophagy of microglia, and the released inflammatory factors further attack neurons (Yang et al., 2015). Based on this mechanism, autophagy inhibitors or Beclin 1 knockout contributes to rescuing transneuronal degeneration of thalamic neurons (Xing et al., 2012). Inhibition of autophagy by 3-MA or wort can also shrink infarct size and protect from oxygen glucose deprivation (OGD)-induced astrocyte damages in rats.

The BBB is composed of endothelial cells, also known as supporting cells, alongside with microglia and pericytes. The BBB maintains homeostasis of the central nervous system (CNS). Post-ICH, the integrity of endothelial cells is impaired, which increases the permeability of BBB and thus induces cerebral edema, leukocyte infiltration, and invasion of neurotoxic and active compounds. Eventually, neuronal loss occurs following the disruption of CNS homeostasis. Endothelial cells are targets of various initiators for apoptosis, including overexpression of Fas ligand, TGF-*β* and p53, deposition of amyloid-β peptide (Aβ) fragments, ATP depletion, increased Bax/Bcl-2 ratio, and caspase cascade (Rizzo and Leaver, 2010). There is a link between vascular endothelial cell apoptosis and neuronal death, and the vascular integrity reflects the degree of neuronal damage (Nagasawa and Kogure, 1989). Neuronal autophagy alleviates endothelial cell damage and hypoxia-induced BBB disruption via inhibiting membranous CLDN5 and CAV1-mediated transcytosis (Yang et al., 2021). Changes in BBB permeability influence the tight junctions between endothelial cells, and the overexpression of tight junction proteins favors to protect BBB permeability. It is reported that overexpression of exosomal miR-124-3p derived by microglia upregulates tight junction proteins, thereafter inducing autophagy but inhibiting apoptosis of endothelial cells (Zhao et al., 2022). Similarly, serum-derived exosomes suppress cell apoptosis in the striatum of rats with cerebral IRI through upregulating Bcl-2/Bax, ZO-1 and claudin-5 and downregulating caspase-3 in the cortex. Differing from the role of exosomal miR-124-3p in inhibiting apoptosis, serum-derived exosomes inhibit autophagy by reducing the LC3B-II/LC3B-I ratio and autophagy flux, thus inhibiting apoptosis (Huang et al., 2022). Astrocyte-derived exosomes consistently protect neurons by suppressing autophagy (Pei et al., 2019). Nevertheless, an excessive autophagy increases BBB permeability via degrading occludin (Kim et al., 2020).

Great efforts have been made for illustrating the transition between autophagy and apoptosis in neurons, microglia and endothelial cells. The distinct expression profiles of autophagic and apoptotic markers at certain time points of ICH models greatly reflect the dual role of autophagy. The autophagy and apoptosis varying across cell types require further *in vivo* experimental mining.

## 6 Autophagy and apoptosis in the treatment of ICH

### 6.1 ICH treatment by Western medicine

Post-ICH hematoma expansion is a risk factor for a poor prognosis, and hematoma volume is considered as a predictor of 30-day mortality and functional disability in ICH patients (Ruiz-Sandoval et al., 2007). As a common approach to treat ICH, surgical removal of hematoma lowers the compression on surrounding tissues and intracranial pressure. For high-risk individuals with large hematomas, either surgery or medication achieves a poor outcome (Gregson et al., 2012).

Tight blood pressure control is the major approach to prevent ICH. An early intensive blood pressure lowering has been recommended to limit hematoma expansion and treat ICH (Li et al., 2020). However, clinical benefits of an early intensive blood pressure lowering after acute ICH remain controversial (Moullaali et al., 2022). Medications are optional to ICH patients with surgical contraindications, including antihypertensive drugs, hemostatic drugs, antioxidants, and anti-inflammatory drugs (Greenberg et al., 2022).

#### 6.1.1 Apoptosis-modulating drugs

Recombinant activated factor VII (rFVIIa) is a natural directly stopping bleeding to treat ICH. rFVIIa inhibit cell apoptosis by enhancing the Bcl-2/Bax ratio in rats with ICH (Ma et al., 2018). Clinical evidence has validated the efficacy of rFVIIa within 4 h of ICH in suppressing hematoma expansion, lowering mortality and improving the prognosis (Mayer et al., 2005).

Edaravone is applied to alleviate ICH-induced cerebral edema, neurological deficits and oxidative stress (Nakamura et al., 2008). It protects against neurological deficits in rats with ICH through downregulating caspase-3 and Bax, and upregulating Bcl-2 (Zhang et al., 2016).

Levetiracetam, widely used to treat epilepsy, exerts the function of protecting neurons post-ICH and preventing secondary injuries (Imai et al., 2020). Animal experiments have shown that levetiracetam inhibits inflammatory response and apoptosis in rats and mice with ICH by regulating the JAK2-STAT3 signaling pathway (Xiong et al., 2020).

Dexamethasone is able to inhibit cell apoptosis by modulating Bcl-2/Bax ratio and downregulating caspase-3, thus protecting against neuronal damage (Lee et al., 2015).

#### 6.1.2 Autophagy-targeted drugs

Ebselen, an autophagy-targeted drug with anti-oxidative properties, alleviates silent brain infarction (SBI) following middle cerebral artery stroke by inhibiting expression levels of LC3-II and Beclin-1 (Li et al., 2015).

Prior to ischemic events, melatonin, a neuroprotective substance, can be used to prevent SBI through inhibiting ER stress-induced autophagy (Feng et al., 2017).

#### 6.1.3 Dual-agents regulating apoptosis and autophagy

Cerebroprotein hydrolysate is a peptidergic drug that uniquely repairs nerve cells. It facilitates the recovery of secondary thalamic damage and neurological function after ICH by downregulating caspase-3 and LC3-II, and enhancing the Bcl-2/Bax ratio to inhibit both apoptosis and autophagy in the ipsilateral thalamus (Xing et al., 2014).

Interleukin 33 (IL-33) is reported to reduce brain water content and thus improves post-ICH neurological function. It executes its neuroprotective role through inhibiting inflammatory responses (downregulation of IL-1β and TNF-*α*), apoptosis (upregulation of Bcl-2 and downregulation of caspase-3) and autophagy (downregulation of LC3-II and Beclin-1) (Gao et al., 2017).

Prevention of SBI is the key strategy to improve the prognosis of ICH. Neuronal excitotoxicity, as an initiator of ICH-induced SBI, can be reduced by α-asarone (ASA) via suppressing mitochondria-mediated apoptosis and neuronal autophagy (Gao et al., 2022).

Lumbrokinase is featured by an antithrombotic activity. It inhibits apoptosis and autophagy by downregulating caspase-12 and NF-κB after cerebral infarction, and also suppresses inflammatory responses via reducing the NLRP3 inflammasome (Wang et al., 2022).

Atorvastatin exerts neuroprotective effects on ICH rats by fighting against apoptosis via downregulating calpain I, CHOP and GRP78 (Chen et al., 2022). Stimulation on autophagy is another mechanism underlying the function of atorvastatin in protecting subarachnoid hemorrhage (SAH) (Chen et al., 2020). Lovastatin inhibits apoptosis and regulates autophagy by silencing the AMPK/mTOR signaling pathway after ICH (Deng et al., 2023).

Minocycline is used to alleviate cerebral edema, maintain the integrity of BBB and improve the prognosis of ICH (Wang et al., 2019). Application of minocycline to ICH/SAH animal models achieves an anti-inflammatory outcome through inhibiting autophagy, apoptosis and release of inflammatory factors and promoting differentiation into M2 microglia (Wu et al., 2016; Dai et al., 2019).

### 6.2 ICH treatment by TCM

#### 6.2.1 Apoptosis-associated TCM components

Baicalein is a flavone originally isolated from the traditional Chinese medicine (TCM) Scutellaria baicalensis. Intraperitoneal injection of baicalein reduces BBB permeability and inhibits cell apoptosis in ICH rats via inhibiting TLR4-mediated MAPK and NF-κB signaling pathways (Wei et al., 2017). Besides, baicalein suppresses apoptosis by inhibiting the Bax/Bcl-2 ratio (Wang et al., 2015).

Curcumin, a beta-diketone constituent extracted from the rhizome of *Curcuma longa*, effectively protects neuroinflammation and neuronal apoptosis by blocking M1 polarization of microglia following ICH (Wang et al., 2022).

Matrine is an alkaloid extracted from the dried roots, plants and fruits of the genus Sophora. It offers an inhibitory effect on inflammatory response, oxidative stress and apoptosis by inactivating the PI3K/Akt-mediated NF-κB signaling pathway and activating the Keap1/Nrf2-HO-1 signaling pathway (Liu et al., 2016).

Oxymatrine is also an alkaloid extracted from the root of *Sophora flavescens*. Similarly, it suppresses neuroinflammation, oxidative stress and neuronal apoptosis through downregulating TLR-4, NF-κB, TNF-*α*, IL-1β, and IL-6 (Huang et al., 2012).

Tetramethylpyrazine, also known as ligustrazine, is a chemical compound with neuroprotective properties. It relieves cerebral edema and inhibits the apoptosis of neurons in the surrounding tissues of hematoma via upregulating p-Akt and Bcl-2/Bax ratio and downregulating caspase-3 (Shao et al., 2018).

Ursolic acid is a pentacyclic triterpene acid widely presenting in loquat leaves, *Ligustrum lucidum* and *Prunella vulgaris* L. Treatment with ursolic acid reduces BBB permeability and brain water content in SAH rats through suppressing inflammatory factors, oxidative stress around hematomas and apoptosis (Zhang et al., 2014).

Ginsenoside Rb1, a triterpenoid saponin extracted from ginseng, favors the recovery of SAH rats by reducing cerebral edema, protecting BBB disruption and inhibiting p53-induced cell apoptosis (Li et al., 2010).

Ligustilide is a lactone compound extracted from Angelica sinensis and Ligusticum wallichii. Through downregulating p53 and caspase-3, ligustilide reduces the number of apoptotic cells in the surrounding area of SAH (Chen et al., 2011).

Triterpenoid saponins isolated from the *Panax pseudoginseng* greatly relieve cerebral edema and inhibit cell apoptosis (Xu et al., 2014).

Puerarin, a phytoestrogen found in the root of Pueraria, suppresses apoptosis and oxidative stress in surrounding brain regions of hematomas via inhibiting the PI3K/Akt-mediated inflammatory response and activating the SIRT signaling pathway (Zeng et al., 2021).

#### 6.2.2 Autophagy-associated TCM components

Luteolin is a natural product found in a variety of edible TCM herbals, including chrysanthemum, *Lonicera japonica* and purple perilla. The neuroprotective function of luteolin post-ICH is dependent on the activated p62/Keap1/Nrf2 signaling pathway to stimulate autophagy and nuclear translocation of Nrf2. Moreover, it enhances autophagy and antioxidant property in both *in vitro* and *in vivo* ICH models, thus protecting against mitochondrial dysfunction in neurons (Tan et al., 2019).

Ginkgetin is a natural non-toxic biflavonoid extracted from the dried leaves of *Ginkgo biloba*. It protects cerebral microbleeds and neurological deficits by mediating autophagy via the JAK/STAT and MAPK signaling pathways (Adnan et al., 2020).

Artesunate is a semi-synthetic derivative of artemisinin. In the ICH rat model, artesunate increases autophagy to treat cerebral infarction via downregulating p-mTOR and upregulating Beclin-1 and Mcl-1 in the ischemic cerebral cortex and primary ischemic hippocampal neurons of rats (Shao et al., 2018).

Hydroxysafflor yellow A, the most effective water-soluble component of safflower, is usually adopted to treat cerebral infarction. It protects the brain after cerebral infarction by downregulating HIF-1, BNIP3, and Notch1 to inhibit cell autophagy (Zhang et al., 2022).

Total phenolics extracted from the lotus plumule protect neurological function after cerebral infarction and reduce cerebral necrosis and infarction by inhibiting autophagy via the PI3K/Akt signaling pathway (Qiao et al., 2023).

#### 6.2.3 Apoptosis-associated TCM compound preparations

A combination of biologically active components of *Ginkgo biloba* greatly promotes the endogenous regression of hematoma by inhibiting neuronal apoptosis post-ICH through suppressing the LR4/NF-κB-dependent inflammatory response (Hu et al., 2011).

Didang Decoction (DDD) is a classic TCM compound preparation composed of rhubarb (Dahuang), peach seed (Taoren), leech (Shuizhi), and gadfly (Mengchong). Its neuroprotective effect in alleviating ICH-induced brain damages and cell apoptosis relies on the inhibition on the GPR78-IRE1/PERK signaling pathway (Huang et al., 2018).

Liangxue Tongyu Formulation, also commonly used to treat ICH, accelerates cell proliferation by activating the PI3K signaling pathway and inhibits glutamate-induced apoptosis of PC12 cells (Li et al., 2018).

#### 6.2.4 Autophagy-associated TCM

Radix astragali-safflower is a classic herbal pair to treat cerebrovascular diseases. It effectively improves neurological function after cerebral infarction and prevents SBI by mediating autophagy through upregulating LC3II/LC3I, Beclin1, and p62 (Chen et al., 2022).

Tongqiao Huoxue Decoction, consisting of Radix Paeoniae Rubra, Ligusticum wallichii, red jujubes, safflower, Zingiberis Rhizoma Recens, Fistular onion stalk and Moschus, pharmacologically promotes blood circulation and dissipates stagnation. It alleviates cerebral ischemic injury by inhibiting autophagy through regulating the PI3K/Akt/mTOR signaling pathway (Hua et al., 2022).

Taohong Siwu Decoction exerts neuroprotective effects on cerebral infarction through upregulating LC3-II/LC3-I, Beclin1, Parkin, and PINK1, thereafter enhancing mitophagy and reducing the NLRP3 inflammasome (Ji et al., 2022).

#### 6.2.5 Dual-regulation of TCM on autophagy and apoptosis

Resveratrol is a natural polyphenol extracted from TCM herbals of Veratrum, *Polygonum multiflorum* and *Polygonum cuspidatum*. Resveratrol reduces cerebral edema post-SAH via regulating both autophagy and apoptosis by the Akt and mTOR signaling pathways (Guo et al., 2018). Through mediating the AMPK/SIRT1 signaling pathway, resveratrol inhibits the activation of microglia and the release of inflammatory cytokines in a SAH model by regulating autophagy (Li and Han, 2018).

Acupuncture is a traditional TCM technique to stimulate hematoma absorption and improve neurological deficits after cerebral hemorrhage (Li et al., 2023). Through downregulating p-mTOR and p-S6K1, acupuncture triggers autophagy and inhibits the mTOR signaling pathway to alleviate neurological deficits in rats at post-ICH (Liu et al., 2022). Scalp acupuncture is a micro-system acupuncture technique that inhibits neuronal apoptosis by upregulating Beclin1, Parkin, PINK1 and NIX protein levels, and downregulating caspase-9 (Liu et al., 2021). Electroacupuncture at the acupoints of GV20 (Baihui)-GB7 (Quyan) enhances mitophagy and inhibits apoptosis after ICH, suggesting the therapeutic efficacy of electroacupuncture on ICH by balancing mitophagy and apoptosis (Guan et al., 2021).

## 7 Discussion

Apoptosis and autophagic cell death are not mutually exclusive; they can occur simultaneously within tissues or even within individual cells. Numerous studies have shed light on the interplay between autophagy and apoptosis at the cellular level. It has been suggested that autophagy may precede apoptosis, acting as an adaptive response to imminent cell death, particularly under conditions such as growth factor deprivation, exposure to toxic agents, hypoxia, and other forms of cellular damage. The lysosome, a crucial organelle in the autophagic process, also plays a regulatory role in apoptosis. Proteases released from lysosomal compartments can initiate apoptotic signaling, and lysosomes are involved in the trafficking of death receptors to the cell surface, influencing cellular sensitivity to external ligands (Bursch, 2001). Both autophagy and apoptosis are essential mechanisms for cellular self-preservation. When cellular injury occurs, apoptosis helps eliminate irreparably damaged cells, minimizing unnecessary energy expenditure. In contrast, autophagy temporarily boosts the energy supply required for cellular functions, mitigating damage and primarily acting as a protective strategy. However, the autophagic degradation of anti-apoptotic proteins can enhance apoptotic activity (Cao et al., 2021). From this perspective, the key factor in autophagy lies in the nature of the autophagic proteins involved. Additionally, autophagy can exacerbate cell death: sustained autophagy due to chronic stress—termed excessive autophagy—can lead to increased apoptosis and even trigger autophagic cell death. Thus, autophagic death and apoptosis are interconnected processes that often occur concurrently (Liu et al., 2023).

While autophagy may seem detrimental following cerebral hemorrhage, macrophages can have a beneficial role in this context, an area that warrants further investigation. Autophagy is also entangled with ferroptosis. Ferroptosis is a new type of non-apoptotic death. After cerebral hemorrhage, iron ions are deposited in the blood. Excessive free iron will produce hydroxyl radicals, leading to cell apoptosis and damage to the blood–brain barrier (BBB) (Wei et al., 2022). Activation of autophagy can promote ferroptosis (Liu et al., 2023). Multiple autophagy factors are involved in ferroptosis, such as NCOA4-promoted ferritinophagy, RAB7A-dependent lipophagy, BECN1-mediated system Xc inhibition, STAT3-induced changes in lysosomal membrane permeability, and HSP90-related chaperones (Xie et al., 2023). Depletion of core components of the autophagic machinery inhibits ferroptotic cell death, such as ATG5, ATG7 and BECN1/Vps30/Atg6 (beclin 1) (Liu et al., 2023). Concurrently, a deficiency in autophagy intensifies the production of reactive oxygen species (ROS) and apoptosis triggered by iron overload, indicating that autophagy plays a protective role in cellular responses to iron overload and oxidative stress (Sung et al., 2023). However, excessive iron overload-induced mitochondrial damage can induce further mitophagy, providing an additional source of iron for lipid peroxidation (Yu et al., 2022). Recent studies have demonstrated that the activation of macrophages can mitigate hematoma formation following cerebral hemorrhage and enhance neurological function. Nonetheless, the release of heme and iron subsequent to erythrocyte rupture may exacerbate ferroptosis by promoting autophagy (Fu et al., 2023)^.^

## 8 Conclusion

Autophagy and apoptosis conjure up a complicated interplay after ICH. Autophagy serves as an initiator and competitor of apoptosis, and also accompanies apoptosis-induced cell death. In the Bcl-2 family, the Bcl-2/Bax ratio not only reflects a state of cell apoptosis, but also cooperates with Beclin 1 to regulate autophagy. ICH-induced apoptosis and autophagy struggle to maintain a dynamic balance. More evidences are needed to illustrate their interactions post-ICH.
