# Supplementary material for: A crosstalk between autophagy and apoptosis in intracerebral hemorrhage
Source: Front Cell Neurosci. 2024 Nov 21;18:1445919. doi: 10.3389/fncel.2024.1445919 (PMC11622039; doi:10.3389/fncel.2024.1445919)
Supplement: Supplementary file 1 [file Table_1.docx]

**Supplementary section**

**1.Experimental Animals**

Sixty-two healthy male Sprague-Dawley adult rats weighing 250±50 g were obtained from the Southwest Medical University Laboratory Animal Center, with Production and Use License numbers SYXK (Chuan) 2023-0017 and SYXK (Chuan) 2023-0065.In order to provide rats with a stable and clean environment for rearing, six rats were housed in cages under specific pathogen-free (SPF) experimental conditions.Throughout the experiment, rats received ad libitum access to a standard diet and clean, filtered water.Every surgical operation was carried out while under 2% sodium pentobarbital anesthesia.Following the alleviation of animal suffering during the experiments, the study was approved by the Animal Ethics Committee of Southwest Medical University. An intraperitoneal injection of 150ug/kg of rapamycin was administered within one hour post-surgery. Rapamycin is dissolved in dimethyl sulfoxide at a concentration of 5 milligrams per milliliter and kept in a freezer at minus twenty degrees Celsius.Rapamycin is diluted in PBS containing 5% Tween 80 and 5% polyethlene glycol 400 .

**2.Grouping and Treatment**

To prepare a cerebral hemorrhage model, anesthetized rats were positioned prone with their heads fixed in a brain stereotaxic apparatus. The apparatus was centered on the rat's coronal suture, and 3% hydrogen peroxide was applied to the periosteum to aid its removal. A 3% hydrogen peroxide solution was used to aid in dissecting the rat periosteum. The microinjector was positioned 1mm anterior to the anterior fontanelle and 3mm from the midline. A dental drill created a 1mm diameter hole. Using stereotactic guidance, a Hamilton syringe with a 26-G needle was inserted into the right deep cortex/basal ganglia at a depth of 5.5 mm at a rate of 1mm/min. The needle was paused for 5 minutes, and the microinjector's height was adjusted and held for 10 minutes. Gently remove the microsampler, fill the needle hole with bone wax, and suture the incision. Disinfect the incision with iodophor wash. Administer 80,000 IU of penicillin intramuscularly daily to each rat to prevent infection. Ensure sterile procedures, and house the rats in separate cages post-surgery. The blank group did not undergo these procedures.

Rats were randomly divided into (1) blank group (Sham): normal rats; (2) cerebral hemorrhage group (ICH): modeling, given an equal amount of saline; (3) Rapamycin group (RAPA): modeling, intraperitoneal injection of 150ug/kg of rapamycin within 1 hour after surgery, given the same amount of saline. All rats were gavaged once a day for 3 consecutive days.

**3.Histological Staining and electron microscopy.**

The brain tissues were sectioned into 4 μm slices and subsequently treated with xylene to remove paraffin, followed by dehydration using alcohol. In the subsequent step, the sections were stained with hematoxylin to facilitate the visualization of brain tissue morphology under a Leica white light microscope (Leica, Germany). Similarly, the brain tissues were stained with Nissl using the same protocol for microscopic observation.

The samples were initially fixed with 3% glutaraldehyde and subsequently post-fixed with 1% osmium tetroxide for electron microscopy analysis. Following dehydration with acetone, the specimens were embedded in Epon812 resin, sectioned using a diamond knife, stained with uranyl acetate and lead citrate, and examined using a transmission electron microscope.

**4.Experiments in Vitro**

SH-SY5Y human neuroblastoma cells, obtained from the Cell Bank/Stem Cell Bank of the Chinese Academy of Sciences, were cultured under controlled conditions in an incubator set at 37°C with 5% CO2. The experimental setup involved seeding the neurons into a 6-well plate, which included the following groups: a control group (Sham) with no treatment; a heme-treated group (Hemin) where heme was administered for 12 hours; an FYXN group receiving both heme and FYXN; and a Rapamycin group (RAPA) treated with both heme and rapamycin.

Cells from each group were subjected to double staining with Bcl-2 antibody, Bax antibody in conjunction with Bcl-2 antibody, and Beclin 1 antibody, followed by observation using a Leica microscope.
